# Can Recurrent Neural Networks Validate Usage-Based Theories of Grammar Acquisition?

**DOI:** 10.3389/fpsyg.2022.741321

**Published:** 2022-03-23

**Authors:** Ludovica Pannitto, Aurelie Herbelot

**Affiliations:** ^1^CIMeC - Centre for Mind and Brain Sciences, University of Trento, Trento, Italy; ^2^Department of Information Engineering and Computer Science, University of Trento, Trento, Italy

**Keywords:** recurrent neural networks, grammar, usage-based linguistics, language acquisition, construction grammar

## Abstract

It has been shown that Recurrent Artificial Neural Networks automatically acquire some grammatical knowledge in the course of performing linguistic prediction tasks. The extent to which such networks can actually learn grammar is still an object of investigation. However, being mostly data-driven, they provide a natural testbed for usage-based theories of language acquisition. This mini-review gives an overview of the state of the field, focusing on the influence of the theoretical framework in the interpretation of results.

## 1. Introduction

Artificial Neural Networks (ANNs), and in particular recurrent architectures such as Long Short-Term Memory Networks (LSTMs) (Hochreiter and Schmidhuber, 1997), have consistently demonstrated great capabilities in the area of language modeling, generating sentences with credible surface patterns and showing promising performance when tested on very specific grammatical abilities (Gulordava et al., 2018; Linzen and Baroni, 2021), without requiring any prior bias towards the syntactic structure of natural languages. From a theoretical point of view, however, published results sometimes appear inconsistent, and overall inconclusive. The present survey suggests however that results should be interpreted in the light of various theoretical frameworks if they are to be fully understood. To illustrate this, it approaches the literature from the point of view of usage-based theories of acquisition, which are naturally suited to the behaviorist setting implemented by language modeling techniques.

## 2. Usage-Based Theories of Grammar Acquisition

Taking a coarse-grained perspective on usage-based theories of language acquisition, we can pinpoint three main standpoints that are relevant to language modeling with ANNs.

First and foremost, behaviorist theories argue for a systemic vision where general-purpose memory and cognitive mechanisms account for the emergence of linguistic abilities (Tomasello, 2003; Goldberg, 2006; Christiansen and Chater, 2016; Cornish et al., 2017). That is, they stand against the idea that explicit, *innate* biases should be required in the acquisition device.

Secondly, usage-based theories argue for a tight relation between *input* and learned representations in the course of acquisition (Jackendoff, 2002; Boyd and Goldberg, 2009). This is based on results that indicate that infants understand and manipulate input signals in sophisticated ways: their ability to analyze stream-like signals like language is well explored in the statistical learning literature (Gómez and Gerken, 2000; Romberg and Saffran, 2010; Christiansen, 2019), and the shape of the input itself has been explained by its relation to basic cognitive processes (Christiansen and Chater, 2015; Cornish et al., 2017). Word segmentation for instance is accomplished by 8-month old infants, relying purely on statistical relationships between neighboring speech sounds, and with very limited exposure (Saffran et al., 1996). Such limited input is also enough for one-year-olds to acquire specific grammatical information, thus discriminating new grammatical strings from those that show string-internal violations (Gomez and Gerken, 1999).

Thirdly, gradedness of grammatical notions is a central aspect in usage-based theories. Cognitive theories tend to blur hard boundaries, e.g. when it comes to the structure of categories (Barsalou, 1987), the content of semantic knowledge (Elman, 2009; McRae and Matsuki, 2009) or the distinction between lexically filled and pattern-like instances (Goldberg, 2006).

Artificial statistical models seem an ideal toolbox to test the above claims. They can be built without hard-coded linguistic biases and they can be fed different types of input to investigate their effect on the acquisition process. Moreover, both their behavior and internal state can be analyzed in various ways. Lakretz et al. (2019) take a physiological approach investigating how, with no explicit bias, specific neurons specialize in detecting and memorizing syntactic structures. Giulianelli et al. (2018) propose instead a diagnostic downstream classifier to evaluate representations of number agreement.

The rest of this survey approaches the literature in the light of the three aspects of usage-based frameworks mentioned above, discussing to what extent the theory fits both implementation and results.

## 3. Neural Language Models and Language Development

The comparison between artificial language models and human language development starts at a fundamental mechanism: prediction. Predictive functions are considered highly relevant to language processing (Pickering and Garrod, 2013; Ramscar et al., 2013) and have received particular attention from theories that posit a direct relation between the shape of the received input and the organization of grammar (Ramscar et al., 2013; Fazekas et al., 2020). Consequently, (artificial) predictive models should be ideally suited to test related hypotheses.

While prediction is a shared mechanisms among neural architectures, different models have been specialized for different tasks, leveraging prediction in various ways. The task most relevant to this survey is known as Language Modeling (LM): networks are trained to *predict* the next word (or character) given the previous sequence. Language Modeling encodes language competence only partially, leaving aside aspects such as interaction, grounding or event knowledge, which are crucial to human linguistic abilities. Nevertheless, it lets us test to what extent grammar can be learned from a pure and linear linguistic signal.

**Figure 1:**
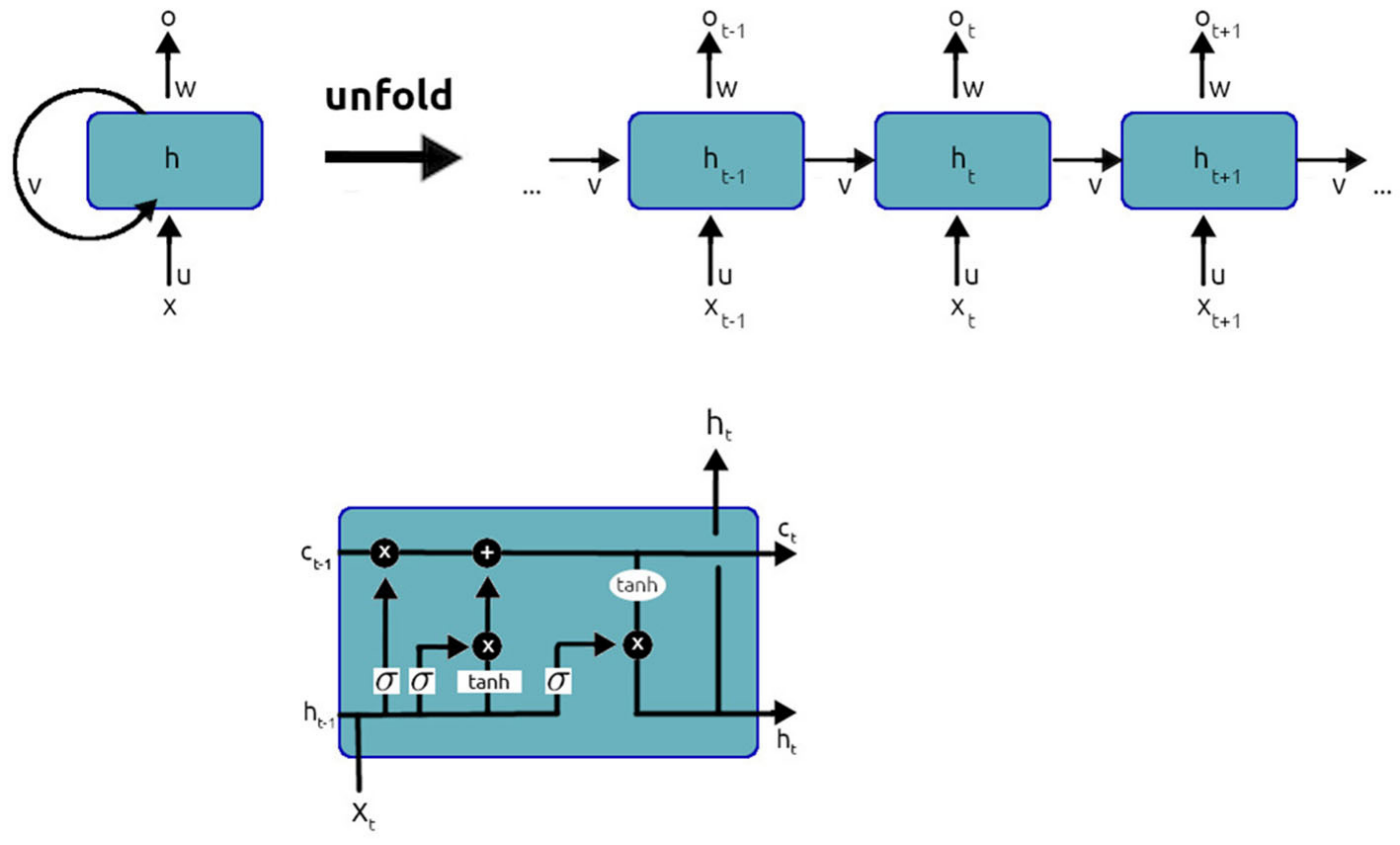
LSTM networks are capable of keeping track of long-term dependencies. As recurrent neural networks (upper layer of the figure), they present a chain-like structure: at each time step t, the network's output is computed based on both the input of time *t*(*x*_*t*_) and the network's state at time *t*−1(*h*_*t*−1_). As opposed to a simple recurrent cell, an LSTM cell (lower layer of the figure) has the ability to regulate how the two kinds of information (input and previous state) are weighted towards the computation of the output. The first gate, the forget gate, evaluates *C*_*t*−1_ (a representation of the previous state different from *h*_*t* − 1_) against *x*_*t*_ and learns what information to keep from previous steps, including it in a vector *f*_*t*_. Next, a candidate value for the current state Ĉ_*t*_ is computed along with the input gate vector *i*_*t*_ that weighs how much of the input will contribute to the current state. Finally, the state of the cell *C*_*t*_ is computed by weighting *C*_*t*−1_ with the forget gate vector *f*_*t*_ and the at Ĉ_*t*_ with the input vector *i*_*t*_. *h*_*t*_ is then computed from *C*_*t*_. A complete and easy to read guide to LSTMs can be found at https://colah.github.io/posts/2015-08-Understanding-LSTMs/.

Recurrent Neural Networks (RNNs), and more specifically the “Long Short-Term Memory network” or **LSTM** (see Figure 1 for a brief description), are among the most common architectures and the ones with the longest history in Language Modeling. In LSTMs, contextual information is maintained from one prediction step to the next. The output of the network at time *t* thus depends on a subset of the inputs fed to the network across a time window. The LSTM learns to regulate its attention over this time window, deciding what to remember and what to forget in the input.

LSTMs are a useful framework to compare learning in a purely predictive setting and an innately biased model. Expectedly, LSTMs that carry explicit syntactic bias [e.g. Recurrent Neural Network Grammars, Dyer et al. (2016); Kuncoro et al. (2017)] and specifically highlight the benefits of top-down parsing as an anticipatory model (Kuncoro et al., 2018) tend to perform better in experiments. But the question asked by usage-based theories is to what extent such hard-coded biases could be learned from language exposure only. A prime example of the pure prediction approach can be found in Gulordava et al. (2018): a vanilla LSTM is trained on a Language Modeling task, under the argument that the predictive mechanism is sufficient for the network to predict long-distance number agreement. The authors conclude that “LM-trained RNNs can construct abstract grammatical representations.” In a more ambivalent study, Arehalli and Linzen (2020) consider how real-time human comprehension and production do not always follow the general grammatical constraint of subject-verb agreement, due to a variety of possible syntactic or semantic factors. They replicate six experiments from the agreement attraction literature using LSTMs as subjects, and find that the model, despite its relatively simple structure, captures human behavior in at least three of them. The authors argue that those phenomena can be regarded as emerging from domain-general processing mechanisms, while also conceding that additional mechanisms might be required to model others.

Notably, LSTMs also process the linguistic signal incrementally, and can be trained on relatively small amounts of data, comparable to the quantities that children are exposed to during the acquisition years (Hart et al., 1997). While this does not make LSTMs plausible models of human cognition, it makes them good benchmarks for building and verifying a range of psycholinguistic hypotheses around incremental processing and the poverty of the stimulus. This feature is especially important to test usage-based ideas that the statistical distribution of child-directed language explains how children acquire constructions in spite of the limited input they receive (see Section 4).

More recently, a new class of models has emerged and shown excellent performance in generating natural language (i.e., Transformer models Vaswani et al., 2017, TLMs) and have in fact been shown to learn structural biases from raw input data (Warstadt and Bowman, 2020). Some psycholinguistic informed approaches have emerged around the architecture. Related the question of acquisition, Warstadt et al. (2020a) and Hu et al. (2020) have compared a range of models, including LSTMs and transformers, on different sizes of corpora. While the amount of training input clearly benefits system performance, Hu et al. (2020) also conclude that the specific hard-coded architecture of a model is more important than data size in yielding correct syntactic knowledge. Their training data is however not characteristic of child-directed input. In contrast, Huebner et al. (2021) focus on training a TLM on developmentally plausible input, matched in quantity and quality to what children are exposed to. The authors also introduce a novel test suite compatible with child-directed language requirements, such as a reduced vocabulary. Their results show that both features of the input and hyperparameters setting are highly relevant for the acquisition process.

While TLMs seem to be a promising new avenue for researchers, they require very large amounts of data to be trained and exhibit a real preference for linguistic generalization, as opposed to surface patterns (Warstadt et al., 2020b). It is also still unclear whether such networks truly generalize or simply memorize patterns they have encountered, leveraging their extremely large size (Kharitonov et al., 2021).

## 4. The role of input

While widely debated in linguistic research, the effect of *input* on learning has received less attention in computational studies, due to the lack of availability of diverse and realistic input data. This aspect is however a pillar of usage-based theories, and can help make sense of various studies that report seemingly inconsistent results across different input data.

Starting with the issue of input size, experiments such as McCoy et al. (2018, 2020) tackle the poverty of the stimulus by testing the acquisition of specific language abilities (i.e., auxiliary inversion). However, the setup in those studies involves no pre-training or Language Modeling phase, therefore treating the phenomenon as a free-standing task. It is difficult to analyze reported results with respect to children acquisition theories, since, as the authors note themselves, humans tend to share processing strategies across phenomena. As mentioned above, Huebner et al. (2021) propose instead an attractive framework tested on TLMs, which is however affected by the exact hyperparameter setting of the model.

Turning to the actual shape of the input, Yu et al. (2020) investigate the grammatical judgments of NLMs in a minimal pair setting (i.e., two sentences that differ in their acceptability due to just one grammatical property). They find that performance is correlated across tasks and across models, suggesting that the *learnability* of an item does not depend on a specific model but seems to be rather tied to the statistical properties of the input (i.e., on the distribution of constituents).

In Davis and van Schijndel (2020), the authors examine biases of ANNs for ambiguous relative clause attachments. In a sentence like *Andrew had dinner yesterday with the nephew of the teacher that was divorced*, both *nephew* and *teacher* are available for modifications by the relative clause: from a purely grammatical perspective, both interpretations are equally plausible. English speakers however have a generic preference for attaching the relative clause to the lower nominal, while other languages such as Spanish show a preference for the higher nominal. RNNs trained on either English or Spanish do not simulate this pattern, and instead consistently prefer the low attachment (similar results are reported in Davis et al. (2020) about the influence of implicit causation on syntactic representations). The authors show this preference is an artifact of training the network on production data which, in Spanish, contains more instances of low attachments. By manually correcting this bias in the input, generating an equal proportion of high and low attachments, they find that a preference for the higher nominal is learnable by the LSTM.

Lepori et al. (2020) experiment with an artificially constructed set of simple transitive sentences (Subject-Verb-Object), containing optional adjectival or prepositional modifiers in a controlled, probabilistic setting. They show that when a BiLSTM is fine-tuned on a distribution which explicitly requires moving beyond lexical co-occurrences and creating more abstract representations, performance dramatically improves: this suggests that a simple sequential mechanism can be enough if the linguistic signal is structured in a way that abstraction is encouraged.

Finally, Pannitto and Herbelot (2020) confirm the tendency of ANNs to reproduce the particular input they are exposed to. They train an LSTM on three different genres of child-directed data. Their results show that when asked to generate, the network accurately reproduces the distribution of the linguistic constituents in its training data, while showing much lower correlation with the distribution of the other two genres.

Overall, there seems to be evidence across the board that the statistical properties of the language input affect learnability as a whole and are responsible for inter-speaker differences. This fits well in a usage-based framework, and it also contributes to a view of grammar that allows for partial competence, as we will now discuss.

## 5. Graded vs. discrete notion of grammar

Usage-based theories take a graded view on acquisition of linguistic structures, acknowledging that partial competence can be observed, blurring the distinction between semantic and syntactic knowledge, and ultimately, allowing for a range of varied grammatical intuitions across speakers. Existing studies on the grammatical abilities of RNNs report results which tend to confirm this view, but they are interpreted in different ways, as we will presently see.

Wilcox et al. (2018) address the phenomenon of filler-gap dependencies (e.g., the dependency existing between *what* and its gap in *I know what/*^⋆^*that the lion devoured - at sunrise*), evaluating the surprisal values assigned by the pre-trained language models of Gulordava et al. (2018) and Chelba et al. (2013). Their results show that neural language models show high peaks of surprisal in the post-gap position, irrespective of the syntactic position where the gap happens (either subject, object or prepositional phrase). When considering the whole clause, however, predictions related to the subject position are much stronger than for the other two positions, correlating with human online processing results. Overall, their results indicate that filler-gap dependencies, and the constraints on them, are acquired by language models, albeit in a graded manner, and in many cases correlate with human judgements. Similar results are reported by Chowdhury and Zamparelli (2018), but the authors commit to a stronger binary distinction between competence and performance, ultimately stating that their model “is sensitive to linguistic processing factors and probably ultimately unable to induce a more abstract notion of grammaticality.”

A call for *full abstraction*, as opposed to a graded view of syntactic abilities, is also expressed in Marvin and Linzen (2018): English artificial sentence pairs (i.e., a grammatical sentence with its ungrammatical counterpart) are automatically built using a non recursive context free grammar, with the intent of minimizing “the semantic or collocational cues that can be used to identify the grammatical sentence.” Two models are evaluated: a simple RNN language model and a multi-task RNN that solves two tasks at the same time, language modeling and a tagging task that superimposes syntactic information, both trained on a Wikipedia subset. Overall, results are varied both between tasks and, for a single benchmark, between different lexical items: a result that, as the authors say “would not be expected if its syntactic representations were fully abstract.” The outcome is however perfectly reasonable in a usage-based framework, if we think of abstraction as induced by the association of specific lexical items with grammatical structure and intentions.

Gradedness is instead the explicit focus of Hawkins et al. (2020), where the authors examine the performance of various pre-trained neural language models, including the LSTM of Gulordava et al. (2018), against a dataset containing human preference judgements on dative alternations in various conditions, manipulating the length and definiteness of the recipient argument. In this study aimed at modeling verb biases, human intuitions are collected and kept as graded values, which the models are tested against. Lexical bias is seen here as a proxy of syntactic abilities rather than as something that might hurt the abstraction process.

Summarizing, we see a growing body of evidence for gradedness of linguistic judgements, both in humans and networks. Interestingly, studies such as Liu et al. (2021) also show that the acquisition of different types of linguistic knowledge proceeds in parallel, but at various rates, in both LSTMs and TLMs. This opens the door for thinking of the potential aggregation of syntactic and semantic knowledge, but also for talking of different levels of competence, as acquisition takes place over time.

## 6. Discussion

The current tendency in the computational community is to give an account of the knowledge acquired at the end of the acquisition process (Linzen et al., 2018, 2019; Alishahi et al., 2019; Baroni, 2020), but the picture emerging from the analysis of NLMs linguistic abilities is variegated, both in terms of approaches and results. To some extent, the inconsistent results reported in the literature are due to differences in theoretical assumptions made by each of the mentioned studies, rather than in experimental designs. As already highlighted by Linzen and Baroni (2021), the conclusions drawn by ANNs studies largely depend on the particular notions of competence, performance, lexicon and grammar that researchers commit to. Perhaps surprisingly, very few studies explicitly link the performance of neural language models to usage-based formalisms.

More specifically, the evaluation of NLMs is widely performed over specialized datasets that capture some highly debated phenomena, such as auxiliary inversion or agreement in increasingly puzzling contexts. Datasets comprehending a wider range of phenomena are now emerging (Hu et al., 2020; Warstadt et al., 2020a). The mastery of such phenomena undoubtedly corresponds to important milestones in acquisition, but they only give a partial view on the learner's trajectory towards full productivity and compositionality. More careful investigations are required to show how biases in the input affect learning and grammatical performance, and how such biases are eventually overcome.

Another issue is that the performance of NLMs is often compared to those of adult speakers. But some usage-based theories rely on the idea that grammar is an ability that evolves throughout the human lifespan, generating different learning patterns in children and adults. To fully explore this idea, studies should increase their focus on alternative datasets, both at input and evaluation stage.

Finally, NLMs are usually treated as an idealized *average* speaker, with their predictions being compared to aggregates of human judgements. While this can be regarded as a necessary simplification, it also mirrors the view that there is a universally shared grammar towards which both speakers and LMs converge, and that this convergence, rather than individual differences, is meaningful. Conceptualizing NLMs as individual speakers rather than communities would probably let different evaluation setups emerge and provide new modeling possibilities for usage-based accounts.

## Author Contributions

LP prepared the literature review. AH supervised the work. LP and AH jointly wrote the survey. Both authors contributed to the article and approved the submitted version.

## Conflict of Interest

The authors declare that the research was conducted in the absence of any commercial or financial relationships that could be construed as a potential conflict of interest.

## Publisher's Note

All claims expressed in this article are solely those of the authors and do not necessarily represent those of their affiliated organizations, or those of the publisher, the editors and the reviewers. Any product that may be evaluated in this article, or claim that may be made by its manufacturer, is not guaranteed or endorsed by the publisher.
